# Overexpression of scavenger receptor and infiltration of macrophage in epicardial adipose tissue of patients with ischemic heart disease and diabetes

**DOI:** 10.1186/s12967-019-1842-2

**Published:** 2019-03-20

**Authors:** Concepción Santiago-Fernández, Luis M. Pérez-Belmonte, Mercedes Millán-Gómez, Inmaculada Moreno-Santos, Fernando Carrasco-Chinchilla, Amalio Ruiz-Salas, Luis Morcillo-Hidalgo, José M. Melero, Lourdes Garrido-Sánchez, Manuel Jiménez-Navarro

**Affiliations:** 1 Department of Endocrinology and Nutrition, Virgen de la Victoria Hospital (IBIMA), Malaga University, Campus de Teatinos s/n, 29010 Malaga, Spain; 2 Centro de Investigación Biomédica en Red de la Fisiopatología de la Obesidad y Nutrición (CIBEROBN), Instituto de Salud Carlos III (ISCIII), Malaga, Spain; 3 Unidad de Gestión Clínica Área del Corazón, Instituto de Investigación Biomédica de Málaga (IBIMA), Hospital Universitario Virgen de la Victoria, Universidad de Málaga (UMA), Campus Universitario de Teatinos, s/n., Malaga, Spain; 4 Centro de Investigación Biomédica en Red Enfermedades Cardiovasculares (CIBERCV), Instituto de Salud Carlos III, Malaga, Spain

**Keywords:** Scavenger receptors, Oxidized low-density lipoprotein, Epicardial adipose tissue, Diabetes mellitus, Ischemic heart disease

## Abstract

**Background:**

Oxidized low-density lipoproteins and scavenger receptors (SRs) play an important role in the formation and development of atherosclerotic plaques. However, little is known about their presence in epicardial adipose tissue (EAT). The objective of the study was to evaluate the mRNA expression of different SRs in EAT of patients with ischemic heart disease (IHD), stratifying by diabetes status and its association with clinical and biochemical variables.

**Methods:**

We analyzed the mRNA expression of SRs (*LOX*-*1*, *MSR1*, *CXCL16*, *CD36* and *CL*-*P1*) and macrophage markers (*CD68*, *CD11c* and *CD206*) in EAT from 45 patients with IHD (23 with type 2 diabetes mellitus (T2DM) and 22 without T2DM) and 23 controls without IHD or T2DM.

**Results:**

*LOX*-*1*, *CL*-*P1*, *CD68* and *CD11c* mRNA expression were significantly higher in diabetic patients with IHD when compared with those without T2DM and control patients. *MSR1*, *CXCL16*, *CD36* and *CD206* showed no significant differences. In IHD patients, *LOX*-*1* (OR 2.9; 95% CI 1.6–6.7; P = 0.019) and *CD68* mRNA expression (OR 1.7; 95% CI 0.98–4.5; P = 0.049) were identified as independent risk factors associated with T2DM. Glucose and glycated hemoglobin were also shown to be risk factors.

**Conclusions:**

SRs mRNA expression is found in EAT. *LOX*-*1* and *CD68* and were higher in IHD patients with T2DM and were identified as a cardiovascular risk factor of T2DM. This study suggests the importance of EAT in coronary atherosclerosis among patients with T2DM.

## Background

Ischemic heart disease (IHD) is a major cause of death and disability in developed countries. Although IHD mortality rates worldwide have declined over the last decades, it persists as responsible for one-third or more of all deaths in adult individuals [1, 2]. Multiple cardiovascular risk factors contribute to the pathogenesis of atherosclerosis [3]. Different strategies have been proposed for improving prognosis (mainly death and hospitalizations) such as percutaneous coronary revascularization, coronary artery bypass surgery and cardiac rehabilitation [4].

In recent years, epicardial adipose tissue (EAT) has been proposed as playing a relevant role in the physiopathology of IHD [5–8]. EAT is located between the myocardium and the serous layer of the pericardium, and in close proximity to the coronary arteries [9]. It was reported that EAT thickness is an indicator of cardiovascular risk [10]. In physiological conditions, EAT participates in the protection of the myocardium and the coronary vessel, maintaining the energy balance. However, dysfunctional EAT has been implicated in the progression and more aggressive course of IHD. One of these pathological conditions which might alter the normal functionality of EAT is the presence of type 2 diabetes mellitus (T2DM) [11, 12]. An altered EAT is able locally to produce reactive oxygen species, cytokines and chemokines which may create a local toxic and proinflammatory environment [13–15]. The inflammation of EAT has been linked to IHD pathophysiology, which can be reflected by increased of macrophage infiltration [16, 17]. In this state, EAT shows a high infiltration of leukocytes [18], specifically T lymphocytes and macrophages [18, 19] and inflammatory cytokines [20].

It is well known that oxidative stress plays an important role in the genesis of T2DM. The increase in systemic oxidative stress seems to be an important mechanism leading to the increase in lipid peroxidation and the oxidative modification of LDL [20]. Oxidized low-density lipoproteins (OxLDLs) play an important role in the formation and development of atherosclerotic plaques and have been associated with most of the proatherogenic risk factors, including obesity, dyslipidemia, metabolic syndrome and T2DM [21–23]. OxLDLs are mainly removed from circulation by a family of membrane bound receptors, called scavenger receptors (SRs). Different classes of SRs, such as Lectin-like Oxidized LDL receptor-1 (LOX-1), CD36, Macrophage scavenger receptor 1 (MSR1), C–X–C motif Chemokine Ligand 16 (CXCL16) and Collectin Placenta 1 (CL-P1) have been identified in various cell types. The expression of these receptors depends largely on the cell type and cell activation, therefore the uptake and subsequent effect of OxLDLs may be different [24]. The presence of these receptors in adipose tissue could be mainly due to the presence of SR in macrophages. However, several studies have shown their presence in adipocytes [25] which could play a role in the metabolism of circulating lipoproteins, including OxLDLs [24, 26, 27].

Based on the evidence mentioned above, the aim of our study was to evaluate the expression of different SRs (*LOX*-*1*, *MSR1*, *CD36*, *CXCL16*, *CL*-*P1*) and the measure of macrophage infiltration (Cluster Differentiation 68 (*CD68*), *CD11c* and *CD206* in EAT in patients with IHD, stratifying by T2DM status. We hypothesized that the mRNA expression of SRs and the infiltration of macrophages in EAT would be different according to the presence of T2DM. We also assessed the possible association between SR expression and clinical and biochemical variables.

## Methods

### Patients

We included 45 patients with IHD who underwent coronary artery bypass surgery (IHD group) and 23 patients without IHD who underwent aortic and/or mitral valve replacement surgery (control group) due this is the only way to obtain EAT in patients without IHD. The group with patients with IHD was divided according to T2DM status: those with T2DM (n = 23) (IHD-T2DM group) and those without T2DM (n = 22) (IHD-NoT2DM group). The IHD group was defined by the presence of at least one coronary stenosis ≥ 50% of luminal diameter by coronary angiogram. The control group had chronic valve heart disease with or without stenosis less than 50% in any vessel requiring valve replacement but not IHD and without T2DM. T2DM was defined as having a history of diabetes diagnosed and/or treated with medication, fasting blood glucose ≥ 126 mg/dL and/or glycated hemoglobin (HbA1c) ≥ 6.5. Diabetic treatment of IHD patients with T2DM was: only diet (n = 6, 26.1%), oral anti-diabetic (n = 12, 52.1%), oral anti-diabetic and insulin (n = 4, 17.4%) and only insulin (n = 1, 4.3%). No patient was taking thiazolidinediones. In the IHD-T2DM patient group, the duration of diabetes was 6.7 ± 2.2 years. Dyslipidemia was defined as having a history of diagnosed and/or treated with medication for elevated triglycerides, low HDL-cholesterol or high LDL-cholesterol. The presence of greater than or equal to 50% luminal diameter stenosis in at least one major epicardial artery by coronary angiogram defined the IHD. Single vessel and multi-vessel disease were defined as the presence of this stenosis in one major epicardial artery and in two or more major epicardial arteries, respectively. Calculation of the Gensini score was initiated by giving a severity score to each coronary stenosis as follows: 1 point for ≤ 25% narrowing, 2 points for 26 to 50% narrowing, 4 points for 51 to 75% narrowing, 8 points for 76 to 90% narrowing, 16 points for 91 to 99% narrowing, and 32 points for total occlusion. Thereafter, each lesion score is multiplied by a factor that takes into account the importance of the lesion’s position in the coronary circulation (5 for the left main coronary artery, 2.5 for the proximal segment of the left anterior descending coronary artery, 2.5 for the proximal segment of the circumflex artery, 1.5 for the mid-segment of the left anterior descending coronary artery, 1.0 for the right coronary artery, the distal segment of the left anterior descending coronary artery, the posterolateral artery, and the obtuse marginal artery, and 0.5 for other segments). Finally, the Gensini score was calculated by summation of the individual coronary segment scores [28]. Patients with coronary disease with lesions that required revascularization surgery and those patients in the control group who had chronic valve heart disease without IHD or DM were included in the study. Moreover, the decision that these patients were included in the study was taken in the clinical session by cardiac surgeons and cardiology experts. Patients with acute inflammatory disease, severe infectious diseases and/or cancer and women who were taking hormone replacement were excluded from the study. All patients gave written informed consent, and the study protocol was approved by the local Clinical Research Ethics Committee and was carried out in accordance with the Declaration of Helsinki.

### Laboratory measurements

Peripheral venous blood was drawn into pyrogen free tubes with or without EDTA as an anticoagulant on the morning of surgery. For serum, the tubes were left at room temperature for 20 min and then centrifuged at 1500*g* for 10 min at 4 °C. Fasting glucose, glycated hemoglobin (HbA1c), basal insulin, total cholesterol, low-density lipoprotein (LDL), high-density lipoprotein (HDL), triglycerides, apoA-1, apoB-100 and C-reactive protein (CRP) were measured in a Dimension autoanalyzer (Dade Behring Inc., Deerfield, IL) in the hospital laboratory. sLRP1 concentration was measured using commercially available enzyme-linked immunosorbent assay (Abbexa, Cambridge, UK) according to the manufacturer’s recommendation. Homeostasis model assessment for insulin resistance (HOMA-IR) score was calculated from fasting insulin and glucose: HOMA-IR = fasting insulin (μIU/mL) × fasting glucose (mmol/L)/22.5. The concentration of OxLDL was measured by a solid phase two site ELISA (Mercodia Developing Diagnostic, Uppsala, SWEDEN). The intra and inter-assay coefficients of variation were 6.4 and 4.7%, respectively.

### Biological material

EAT biopsy samples (average 0.2 to 0.5 g) were obtained with a precise surgical technique during the heart surgery, near the proximal right coronary artery and located between the myocardium and the visceral pericardium. Pericardial adipose tissue is located on the external surface of the parietal pericardium and is vascularized from non-coronary sources [29]. The sample was collected approximately 1 h after the beginning of general anesthesia. All the tissues were washed in physiological saline, immediately frozen in liquid nitrogen and maintained at − 80 °C until RNA analysis.

### RNA extraction and real time quantitative transcription polymerase chain reaction

Total RNA from frozen human EAT was isolated using RNeasy Lipid Tissue Mini Kit (Qiagen, GmbH, Germany) as previously described [30–32]. Total RNA was reverse transcribed using random hexamers as primers and transcriptor reverse transcriptase (Roche, Mannheim, Germany). Gene expression was assessed by real time PCR using an Applied Biosystems 7500 Fast real time polymerase chain reaction system (Applied Biosystems, Darmstadt, Germany). Reactions were carried out in duplicate for all genes using specific TaqMan^®^ Gene Expression Assays: macrophage scavenger receptor 1 (*MSR1*) (Hs00234007_m1, RefSeq. NM_002445.3, NM_138715.2, NM_138716.2), chemokine (C–X–C motif) ligand 16 (*CXCL16*) (Hs00222859_m1, RefSeq. NM_001100812.1, NM_022059.2), oxidized low-density lipoprotein (lectin-like) receptor 1 (*LOX*-*1*) (Hs01552593_m1, RefSeq. NM_001172632.1, NM_001172633.1, NM_002543.3), collectin sub-family member 12 (*CL*-*P1*) (Hs00560477_m1, RefSeq. NM_130386.2), *CD68* (Hs00154355_m1, RefSeq. NM_001040059.1, NM_001251.2), integrin subunit alpha X (*CD11c*) (Hs00174217_m1, RefSeq. NM_000887.4, NM_001286375.1), mannose receptor, C type 1 (*CD206*) (Hs00267207_m1, RefSeq. NM_00267207_m1), CD36 (Hs01567185_m1, RefSeq. NM_000072.3, NM_001001547.2, NM_001001548.2, NM_001127443.1, NM_001127444.1, NM_001289909.1, NM_001289911.1) and LDL receptor related protein 1 (*LRP1*) (Hs00233856_m1; RefSeq. NM_002332.2). During PCR, the threshold value for all genes studied was 0.1. The Ct value for each sample was normalized by constitutively expressed cyclophilin A signals (*PPIA)* (4326316E, RefSeq. NM_021130.3). SDS software 2.3 and RQ Manager 1.2 (Applied Biosystems, Foster City, CA) were used to analyze the results with the comparative Ct method (2^−ΔCt^). All data were expressed as an n-fold difference relative to the calibrator.

### Statistical analysis

Statistical analyses were performed with SPSS for Windows version 15 (SPSS Inc. Chicago, IL, USA). The normality of continuous variables was checked by means of the Kolmogorov–Smirnov test. Continuous variables are summarized as mean ± standard deviation, and as mean ± SEM in figures. Discrete variables are summarized as frequencies (percentages). The comparison between the results of the different groups was made with the Chi square test and Fischer exact test for categorical data, or with the analysis of variance (ANOVA) and Kruskal–Wallis for continuous variables. Logistic regression models were used in order to identify independent factors (odds ratio [OR]; 95% confidence interval) for IHD in patients with T2DM associated with mRNA expression of SRs, as well as to control for confounding factors. The inclusion of 7 patients in each group for an α of 0.05 (assuming a difference of 0.22 of CD36 mRNA expression, a standard deviation of 0.14 and a power of 0.80) has the power to detect a significance difference of 95%. Values were considered to be statistically significant when P < 0.05.

## Results

### Clinical and biochemical characteristics of the patients

A total of 68 patients were included in our study. Among the 45 patients of the IHD Group, 23 (51.1%) had T2DM (IHD-T2DM group). Table 1 summarizes the anthropometric, clinical and biochemical characteristics of the IHD groups and controls. No significant differences were found between the groups regarding age, gender, cardiovascular risk factors and left ventricular ejection fraction. However, IHD-T2DM patients showed significantly higher levels of fasting glucose, HbA1c, insulin, HOMA-IR score and C-reactive protein than IHD-NoT2DM and controls. The levels of oxLDL and LRP1 were significantly higher in IHD-T2DM patients than in control group.

**Table 1:** Clinical and biochemical characteristics of patients with ischemic heart disease according to the presence of type-2 diabetes mellitus and control group

Variables	IHD-T2DM (n = 23)	IHD-NoT2DM (n = 22)	Control (n = 23)	P value*	P value**	P value***	P value
Age (years)	64.2 ± 10.1	65 ± 10.8	62 ± 10	0.451	0.302	0.196	0.298
Male gender	18 (78.3%)	18 (81.8%)	15 (65.2%)	0.530	0.257	0.257	0.467
BMI (kg/m^2^)	28.6 ± 4.6	28.5 ± 4.5	27.8 ± 4.1	0.734	0.675	0.410	0.689
Smoking	17 (73.9%)	16 (72.7%)	12 (52.2%)	0.545	0.352	0.373	0.498
Obesity	13 (56.5%)	11 (50%)	10 (43.4%)	0.362	0.197	0.456	0.241
Hypertension	20 (86.9%)	18 (81.8%)	17 (74%)	0.314	0.176	0.422	0.217
Dyslipidemia	19 (82.6%)	18 (81.8%)	16 (69.6%)	0.574	0.117	0.176	0.196
Cerebrovascular disease	2 (8.7%)	1 (4.5%)	2 (8.7%)	0.500	0.744	0.482	0.485
Cardiovascular disease history	23 (100%)	22(100%)	2 (8.7%)	0.687	0.035	0.040	0.048
Cardiovascular disease family history	6 (26.1%)	3 (13.6%)	1 (4.3%)	0.146	0.078	0.357	0.176
Left ventricular ejection fraction (%)	55 ± 6	53 ± 6	52 ± 6	0.352	0.146	0.397	0.284
Left ventricular ejection fraction ≤ 40%	5 (21.7%)	4 (18.2%)	4 (17.4%)	0.112	0.115	0.651	0.209
Framinghan score	14.4 ± 8.1	16.8 ± 9.5	9.6 ± 5.8	0.546	0.112	0.048	0.093
Medications, n (%)							
Statin	12 (52.2)	8 (36.4)	9 (39.1)	0.354	0.346	0.850	0.423
ACEI/ARB	10 (43.4)	5 (22.7)	6 (26.1)	0.231	0.205	0.796	0.215
Beta-blocker	14 (60.9)	8 (36.4)	15 (65.2)	0.129	0.922	0.076	0.095
Biochemical data							
Glucose (mg/dL)	167 ± 31	100 ± 29	100 ± 29	< 0.001	< 0.001	0.919	< 0.001
HbA1c (%)	7.9 ± 1	5.7 ± 0.5	5.5 ± 0.5	< 0.001	< 0.001	0.186	< 0.001
Insulin (μIU/mL)	19.6 ± 15	7 ± 4.4	9.4 ± 5	0.133	0.215	0.500	0.187
HOMA-IR	10.3 ± 8.3	1.7 ± 1	2.5 ± 1.6	0.133	0.111	0.527	0.111
Cholesterol (mg/dL)	163 ± 31	157 ± 28	150 ± 29	0.641	0.108	0.158	0.199
LDL cholesterol (mg/dL)	101 ± 21	99 ± 19	103 ± 21	0.910	0.925	0.970	0.899
HDL cholesterol (mg/dL)	34 ± 6	41 ± 9	44 ± 9	0.033	0.001	0.427	0.004
Triglycerides (mg/dL)	189 ± 59	169 ± 41	148 ± 40	0.202	0.004	0.133	0.014
ApoB-100/ApoA-1	0.523 ± 0.101	0.743 ± 0.230	0.757 ± 0.240	0.267	0.222	0.998	0.287
OxLDL (units/L)	63.4 ± 10	54.4 ± 5.3	51.0 ± 8.6	0.118	0.033	0.472	0.081
LRP1 (ng/mL)	0.925 ± 0.438	0.677 ± 0.493	0.789 ± 0.267	0.093	0.036	0.257	0.067
CRP (mg/dL)	71.6 ± 42	27.3 ± 19	19.2 ± 22	0.138	0.056	0.354	0.104
Characteristics of coronary disease							
Multivessel coronary disease	19 (82.6%)	13 (59.1%)		0.01			
> 50% stenosis LMA	16 (69.6%)	10 (45.5%)		0.01			
> 50% stenosis ADA	22 (95.7%)	16 (72.7%)		0.01			
> 50% stenosis CA	18 (78.3%)	13 (59.1%)		0.01			
> 50% stenosis RCA	19 (85.6%)	14 (63.6%)		0.01			
Gensini score	45 ± 14	34 ± 12		< 0.001			

Values are shown as mean ± SD and frequencies (percentages)

ADA, anterior descending artery; CA, circumflex artery; IHD-DM, ischemic heart disease-diabetes mellitus; IHD-NDM, ischemic heart disease-non diabetes mellitus; CRP, C-reactive protein; BMI, body mass index; HbA1c, glycated hemoglobin; HDL, high-density lipoprotein; HOMA-IR, homeostasis model assessment for insulin resistance IU/L, international units/liter; kg/m^2^, kilogram/square metre; LDL, low-density lipoprotein; LMA, left main artery; mg/dL, milligram/deciliter; mmol/L, milimol/liter; ACEI, angiotensin converting enzyme inhibitor; ARB, angiotensin II receptor blocker; OxLDL, oxidized low-density lipoprotein; LRP1, low density lipoprotein receptor-related protein 1; RCA, right coronary artery

P value: overall comparison for all groups. * P value: IHD-T2DM vs. IHD-NoDM; ** P value: IHD-T2DM vs. control group; *** P value: IHD-NoDM vs. control group

On the other hand, the characteristics of coronary artery disease were significantly different between the IHD groups. IHD-DM patients were more likely to have multi-vessel disease and major coronary stenosis than the IHD-NoT2DM patients (Table 1). Also, patients with T2DM had more severity of coronary disease measured by the Gensini score compared with those without T2DM (Table 1).

### mRNA expression of SRs and infiltration of macrophage in EAT

**Fig. 1:**
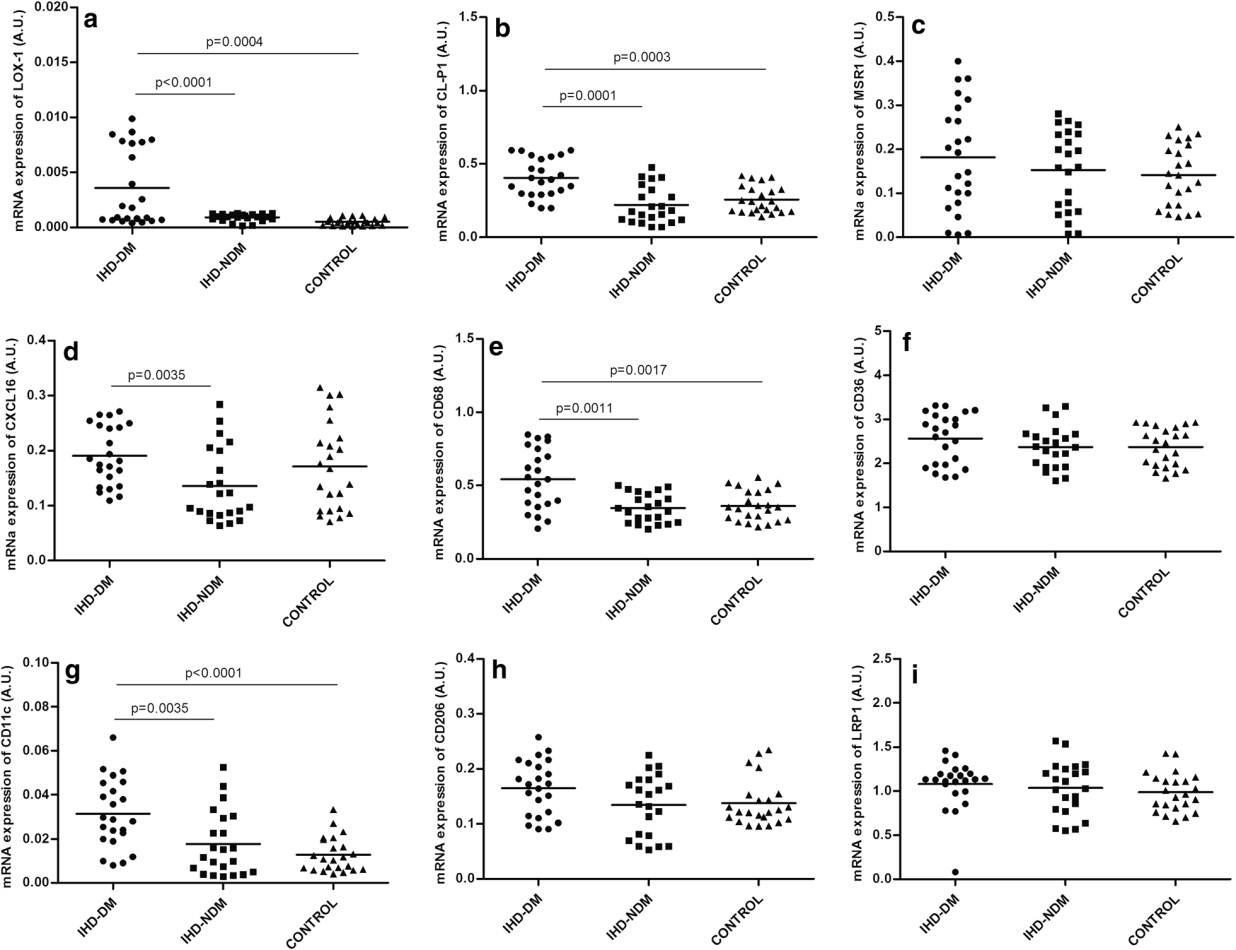
Levels of mRNA expression of SRs, macrophage infiltration and LRP1 in human epicardial adipose tissue in the three groups of patients. **a** Levels of mRNA expression of *LOX*-*1*. **b** Levels of mRNA expression of *CL*-*P1*. **c** Levels of mRNA expression of *MSR1*. **d** Levels of mRNA expression of *CXCL16*. **e** Levels of mRNA expression of *CD68*. **f** Levels of mRNA expression of *CD36*. **g** Levels of mRNA expression of *CD11c*. **h** Levels of mRNA expression of *CD206*. **i** Levels of mRNA expression of *LRP1*

The mRNA expression of SRs in EAT in the three groups of patients is shown in Fig. 1. The mRNA expression of *LOX*-*1*, *CL*-*P1*, *CD68* and *CD11c* in EAT were significantly higher in IHD-T2DM patients when compared with IHD-NoT2DM (P < 0.01) and control patients (P < 0.01) (Fig. 1a, b, e and g, respectively). However, mRNA expression of *MSR1*, *CXCL16*, *CD36*, *CD206* and LRP1 showed no significant differences (Fig. 1c, d, f, h and i, respectively). The mRNA expressions of SRs were similar in IHD-NoT2DM and control patients (Fig. 1).

**Fig. 2:**
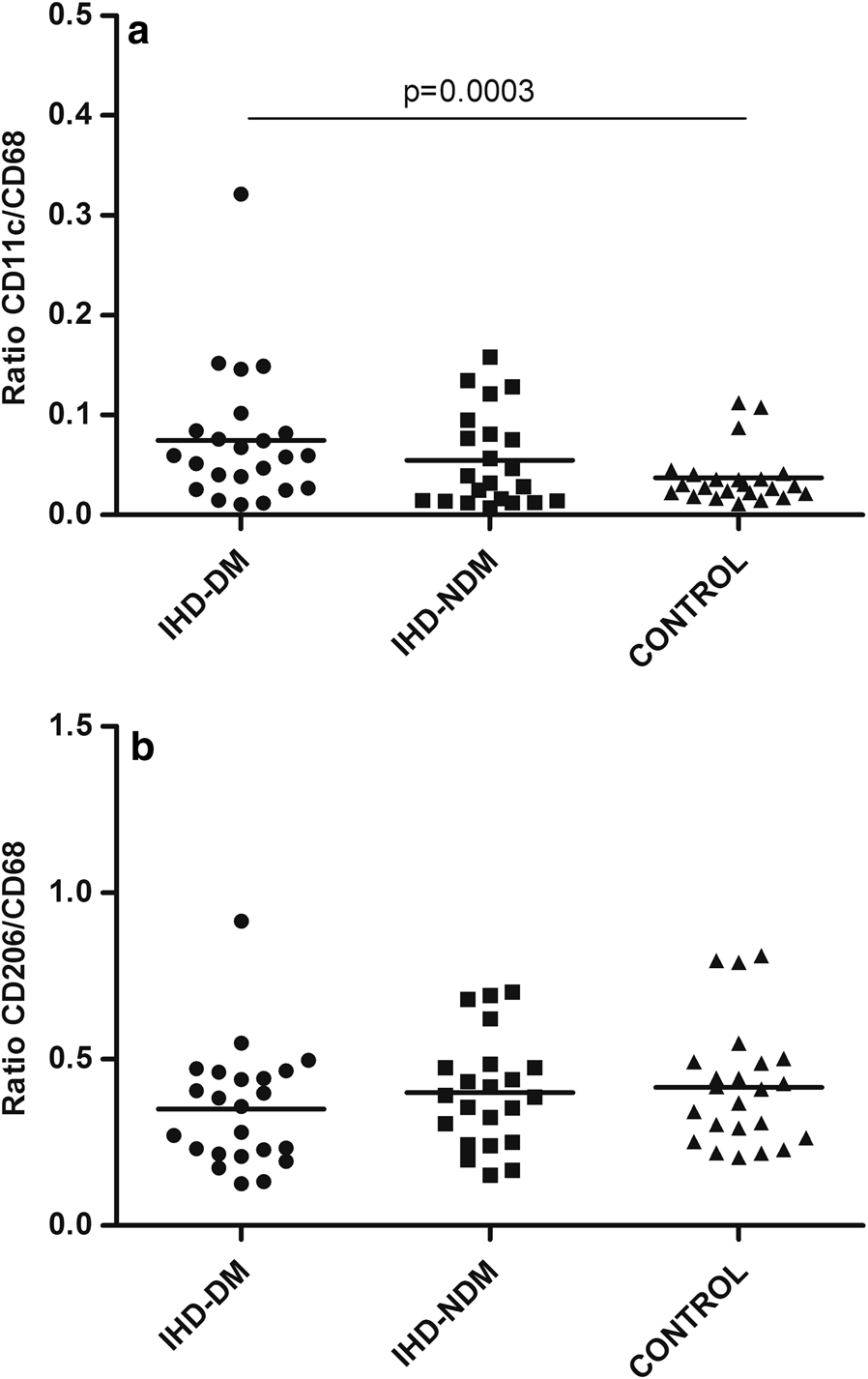
Ratio of *CD11c/CD68* and *CD206/CD68* in human epicardial adipose tissue in the three groups of patients. **a** Ratio of *CD11c/CD68*. **b** Ratio of *CD206/CD68*

With respect to the distribution of M1 and M2 macrophages, significant differences were found in *CD11c/CD68* ratio (M1 macrophages) between IHD-T2DM and control group, showing a higher level those IHD-DM patients (Fig. 2a). No significant differences were found in the *CD206/CD68* ratio (M2 macrophages) (Fig. 2b).

### Associations in EAT between the mRNA expression of SRs and the presence of T2DM in patients with IHD

The factors associated with the presence of T2DM in patients with IHD in a logistic regression model was the level of *LOX*-*1* and *CD68* mRNA expression in EAT. These receptors were identified as independent risk factors of suffering T2DM. Glucose and HbA1c were also shown to be risk factors. This regression was adjusted for sex, age, BMI, statins, FRS and CL-P1 mRNA expression (Table 2). No significant association was found between LOX-1 and T2DM duration.

**Table 2:** Factors associated with type 2 diabetes mellitus in patients with ischemic heart disease

VARIABLE	OR (95% CI)	P value	B coefficient
Glucose	5.9 (2.4–9.0)	< 0.01	0.688
HbA1c	4.0 (1.9–8.1)	< 0.01	0.621
*LOX*-*1*mRNA (AU)	2.9 (1.6–6.7)	0.019	0.599
*CD68* mRNA (AU)	1.7 (0.98–4.5)	0.049	0.531

OR (95% CI) and B coefficient are shown. Logistic regression analysis for T2DM in patients with IHD

*LOX*-*1*, Lectin-like Oxidized LDL receptor-1; *CD68*, cluster of differentiation 68; HbA1c, glycated hemoglobin; OR, odds ratio; 95% CI, 95% confidence interval; AU, arbitrary units

## Discussion

The main finding of our study was that the mRNA expression of SRs is expressed in EAT and this expression was significantly higher for *LOX*-*1* and *CL*-*P1* in IHD-T2DM patients. On the other hand, we found that the infiltration of macrophages was enhanced in the EAT of IHD-T2DM patients, when compared with the IHD-NoT2DM and control group. In addition, the expression of LOX-1 and CD68, glucose and HbA1c levels were identified as risk factors of suffering T2DM in patients with IHD.

To our knowledge, this is the first research study to analyze the expression of SRs (*LOX*-*1*, *MSR1*, *CD36*, *CXCL16* and *CL*-*P1*), *CD68*, *CD11c* and *CD206* in EAT in patients with IHD, stratifying by T2DM status. Our findings add to the limited number of studies that have reported the role of this adipose tissue in coronary atherosclerosis. There is limited existing research proposing EAT as a player in the physiopathology of coronary atherosclerosis [5–8]. Moreover, the presence of SRs in visceral adipocytes has not been widely described [24, 26, 33].

LOX-1 is one of the main SRs for OxLDL [34–36]. Under physiological conditions, these receptors are almost undetectable, however, under exposure to several proinflammatory and proatherogenic stimuli, such as diabetes, hypertension, and dyslipidemia, they are overexpressed [35]. In our study, we showed an overexpression of *LOX*-*1* in EAT among patients with IHD and T2DM compared with IHD without T2DM and control group, suggesting that this adipose tissue which is anatomically in direct contact with the heart and coronary arteries, could be linked to coronary atherosclerosis. Our results showing an association between *LOX*-*1* expression and T2DM and glucose agree with previous studies in which *LOX*-*1* expression was induced by high glucose levels, which seems to be NADPH oxidase-dependent [37]. In line with our findings, the overexpression of *LOX*-*1* in several tissues has also been related with the development and progression of T2DM and its cardiovascular complications [38, 39]. The role of *LOX*-*1* in myocardial ischemia has been shown in some reports. Li et al. [40] showed up-regulated *LOX*-*1* levels in the heart after a short period of coronary artery occlusion, associating with markers of inflammation, oxidative stress, and apoptosis. Similarly, Lu et al. [41] studied the modulation of myocardial damage and heart function induced by permanent coronary occlusion and found that the *LOX*-*1* gene deletion improved survival in mice. In addition, *LOX*-*1* has been also implicated in the collagen deposition after myocardial ischemia, favoring cardiac remodeling [35, 41]. Its gene deletion importantly reduced the process of cardiac remodeling and scar formation, preserving ventricular ejection fraction in mice [41].

With respect to the macrophage infiltration, our work has shown that mRNA expression of *CD68* was increased in EAT in patients with IHD and T2DM when compared with patients without T2DM and control group. In this sense, there are studies that have shown an increase in macrophage infiltration in the EAT of CAD patients, reflected by increased *CD68* macrophage infiltration [16, 17]. In recent years, several studies have indicated that insulin resistance and T2DM are associated with the inflammation of adipose tissue [42–44]. However, limited studies have focused on the inflammatory profile of EAT and its possible involvement with atherosclerosis [6, 11, 43]. In harmony with our findings, Bambace et al., determined concomitantly mRNA expression levels of *CD68* in both subcutaneous and epicardial adipose tissue in male patients with and without T2DM and observed higher *CD68* gene expression levels in both tissue types in diabetic patients than in those without T2DM [43]. Moreover, when we study the distribution of macrophage subtypes, our study shows that only *CD11c* and *CD11c/CD68* ratio (M1-macrophage phenotypes), but not *CD206*, were more significantly overexpressed in IHD-T2DM patients when compared to IHD-NDM or control group. In agreement with our findings, Gurses et al. [16] observed a shift to pro-inflammatory M1-macrophage phenotype in EAT of patients with coronary artery disease (CAD) compared to the control group (without CAD), reflected by increased of *CD11c*, *CD11c/CD68* and *CD11c/CD206* ratios, but not *CD206*. According to our results, Hirata et al. [17] showed similar results, demonstrated that pro-inflammatory macrophages are more dominant in EAT when compared with and without CAD patients. Therefore, our findings suggest that infiltration of macrophages could also cause local inflammation in EAT and these cells could leak free fatty acids. Also, the increase of macrophage infiltration seen in the T2DM patients is consistent with the increase of SRs expression in EAT. Moreover, we have found that macrophage infiltration and these SRs are associated with T2DM independently of BMI, factor directly involved in the adipose tissue inflammation. Our findings would suggest that is not just obesity and BMI that explains this relationship. In this sense, in a recent study, Antonopoulos et al. [45] show that adipose inflammation may contribute to atherosclerosis.

Regarding the mRNA expression levels of *CL*-*P1*, we described for the first time that *CL*-*P1* is expressed in EAT and its expression is significantly greater in IHD-T2DM than in those patients without T2DM and controls. This finding could be related to the function of this SR, mediating the uptake of Ox-LDL and collaborating with other SRs in coronary atherosclerosis. However, its involvement in atherosclerosis has not been clearly described. CL-P1 plays a key role in host defense [46]. In a recent report, CL-P1 has been proposed as inhibiting complement activation and host damage in order to protect self-tissues in acute phase responses [47]. Its expression has been shown in human and murine vascular endothelial layers but its proangiogenic role has not been specifically described [48].

*MSR1* and *CXCL16* were also expressed in EAT, showing higher expression levels in diabetic patients with IHD than in those without T2DM and controls, however, the differences were not significant. Although SRs were originally identified by their ability to recognize and to remove OxLDL, they are very versatile, with a large repertoire of functions such as the elimination of physiological and microbial substances, a critical role in the innate immunity, lipid transport and tissue homeostasis. This wide heterogeneity determines the implication of these receptors in the pathogenesis of different diseases [49] and could explain the differences in the expression levels of these receptors.

Everyday increasing number of diabetic patients and CAD are managed in hospitals and the number will be epidemic in next years due mainly to increase life expectancy and levels of obesity [50]. Proposed strategies achieve a clinical improvement to a certain level [51, 52] and new clinical scenarios such as incomplete revascularization are described [53]. Knowing exactly mechanisms that underlie us importance of EAT can help us to develop new therapeutic strategies and to be able to improve the prognosis of diabetic patients with coronary disease.

We would like to acknowledge some limitations of this study, which is descriptive and no mechanistic insight is provided to e.g. explain the increase in SRs expression in EAT or to identify the cells which express SRs in EAT. Also, this is not a study entirely new. Earlier reports have already described recruitment of macrophages to EAT [18, 19], with an increase in T2DM patients [43]. Moreover, we recruited a relatively small number of patients; our data were from a single hospital and only small samples of EAT were collected from each patient, being insufficient for a thorough analysis and correlation between mRNA expression and protein. Due to the small amount of EAT sample obtained from each patient, we could not measure CD31, as a marker of endothelial cells, and *SCARB1* or ABCA1, involved in the regulation of cholesterol accumulation. Also, another limitation of the study is that we did not perform any specific cardiac test, out of our routine clinical practice, to measure the volume of EAT. EAT measurement requires experts trained specifically in cardiac imagination to obtain valuable data. However, our study was carried out using a well-designed protocol and well established methods. Finally, our study reveals an association, however not a clear causal relationship. The hypothesis that the SRs mRNA expression in EAT is different according to the presence of diabetes and that it could be involved in the pathophysiology of coronary atherosclerosis would need to be confirmed in further research. Therapeutic targeting to EAT regarding the SRs is one choice and this discovery-type of study by the authors would really need repeated investigations using other independent sample sets.

## Conclusions

Our data revealed a predominantly inflammatory profile in EAT in diabetic patients with IHD in comparison with those without DM and controls, showing the implication of *LOX*-*1* as the main SR expressed in EAT and infiltration of macrophage.

SR genes are expressed in EAT. *LOX*-*1* and *CD68* were higher in ischemic heart disease with T2DM than in those patients without T2DM and control patients, and were associated as a cardiovascular risk factor of ischemic heart disease and the severity of CAD, suggesting the importance of EAT in the coronary atherosclerosis among patients with T2DM.
